# Physiology and Pathophysiology 2023

**DOI:** 10.1093/function/zqad005

**Published:** 2023-02-01

**Authors:** Ole H Petersen

**Affiliations:** School of Biosciences, Sir Martin Evans Building, Cardiff University, Cardiff CF10 3AX, UK

**Figure 1.**
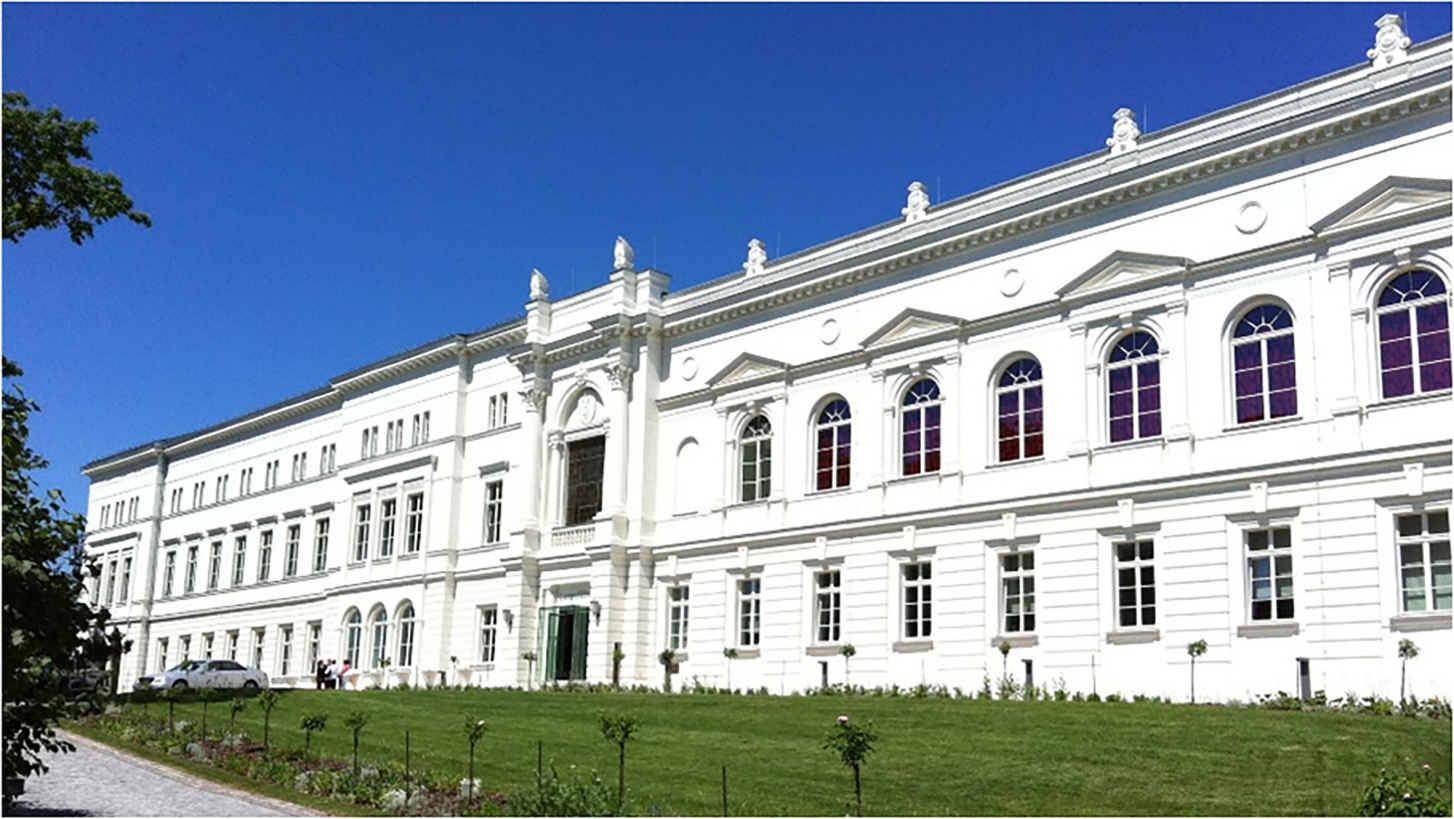
The German National Academy of Sciences Leopoldina (founded in 1652), Halle, Germany, where the symposium on “Physiology and Pathophysiology 2023” will take place on March 7 and 8, 2023.

“Physiology and Pathophysiology 2023” is the name we have given to the conference, organized by the American Physiological Society’s *Function*, the German National Academy of Sciences Leopoldina, and Academia Europaea, to be held at the Leopoldina Academy in Halle, Germany (Figure 1) on March 7 and 8, 2023. On the flyer announcing the program, I had written the following motivation for the symposium: “By investigating organelles, cells, organs and organisms, physiologists work to define the mechanistic basis of living systems in health and disease. However, in spite of major recent advances, it is a serious problem that the biomedical sciences are currently ‘drowning’ in masses of data that do not generate new ideas or models. We are in danger of missing the ‘big picture’. The conference will therefore be a very broad physiology/pathophysiology symposium that takes us all the way from the main bodily support systems to the principal functions, namely, sensing, moving, talking, and thinking. We aim for presentations that provide a broad perspective without getting lost in minor details and there will be adequate time for meaningful discussions.”

Although many are unwilling to admit it, science is driven by irrational fashions. Physiology has for a long time been suffering from some of these trends. Physiologists working on the nervous system, for example, no longer want to be known as neurophysiologists, but have renamed themselves neurobiologists or neuroscientists. These names are, for some reason, regarded as more fashionable. It has also been suggested that physiologists should rename themselves system biologists. The great molecular biologist Sydney Brenner, in his column “Loose Ends,” published regularly in *Current Biology* in the 1990s, wrote in one of his pieces^1^ that: “There is another subject that disappeared a few decades ago which we also need to reinvent, and that is physiology.” The apparent arrogance implicit in this statement, namely that molecular biologists know more about physiology than physiologists, may reflect the notion that molecular biology is more basic than physiology and therefore physiology needs to be seen as one of its subdivisions. Here, it is relevant to consider what Martin Rees, in his recent book “If Science Is To Save Us,”^2^ has to say about the relationships between different scientific disciplines: “It’s a standard metaphor to liken the different sciences to successive levels of a tall building: physics on the ground floor, then chemistry, then cell biology, and all the way up to psychology—with the economists in the penthouse. There is a corresponding hierarchy of complexity: atoms, molecules, cells, organisms, and so forth. But the analogy fails in a crucial respect. In a building, insecure foundations imperil everything above, but the ‘higher level’ sciences dealing with complex systems aren’t imperilled by an insecure base. The uncertainties of subatomic physics are irrelevant to biologists and environmentalists.” Much, but certainly not all, of the great edifice of contemporary physiology is independent of the details of molecular biology.

The borderlines between different biological disciplines are no longer as clear as they appeared to be when I started my research work in the 1960s. At that time, physiologists would often talk with contempt about how boring anatomy was. This followed a long history of competitive antagonism. When I took up the Symers Chair of Physiology at the University of Dundee in 1975, I found papers and pictures in my office, which had once belonged to Waymouth Reid^3^ (Symers Professor of Physiology, 1889–1935), showing that he occasionally hanged an effigy of the professor of anatomy on a miniature gallow at the end of his lectures to medical students. However, anatomy—and specifically light microscopy—has experienced a remarkable renaissance. Results of “dead morphology” on fixed tissues is increasingly being supplemented, and even replaced, by direct observations on living cells in relatively intact preparations.^4^ Imaging of living tissues, even in moving animals,^5^, ^6^ has become one of the essential techniques for contemporary physiology.

In contrast to Sydney Brenner’s opinion that physiology has disappeared,^1^ one could argue that physiological research followed up by physiologically based pharmacology may have had a greater positive impact on the health and welfare of the world’s population than any other branch of biology. In my own field of gastroenterology, there is an impressive example. Through basic physiological work, investigating the mechanism by which gastric acid secretion occurs, George Sachs^7^ and John Forte^8^ identified the H^+^/K^+^ ATPase pump in the secretory canaliculus of the oxyntic cell as the key transporter. The search for pharmacological inhibitors of this pump, by Sachs and his collaborators, was successful,^9^ and the effective proton pump inhibitor (PPI) omeprazole was launched in Europe in 1988 and in the USA in 1990. It revolutionized the treatment of peptic ulcer and gastro-esophageal reflux disease, which in my time as a medical student at the University of Copenhagen (1960s) was by far the most frequent cause of hospitalization in gastro-intestinal wards and could not be treated properly. In the late 1990s, omeprazole became the world’s best-selling drug. A further refined PPI, esomeprazole, can now be found on the shelves of most supermarkets worldwide. It is so safe that it can be bought without prescription and there is not even a requirement to talk to a pharmacist. By solving the problem of treating a very common, immensely painful, and dangerous disease effectively, rapidly, and safely, George Sachs did more for the quality of life, particularly of elderly people, than any other biomedical scientist I have known. His work also exemplifies that there can be no valid pathophysiology and medicine without a proper knowledge of basic physiology.

Because of the astonishing speed with which science has recently advanced, one might think that a time will come when we’ll know it all. The great mathematician and physicist Freeman Dyson addressed this question in his challenging book^10^ “The Scientist as Rebel”: “Science has three advancing frontiers that will always remain open. There is the mathematical frontier, which will always be open thanks to Gödel (Gödel gave examples of undecidable statements that cannot be proved true or false using the normal rules of logic and arithmetic. His theorem implies that mathematics is inexhaustible.). There is the complexity frontier, which will always remain open because we are investigating objects of ever-increasing complexity, molecules, cells, animals, brains, human beings, and societies. And there is the geographical frontier, which will always remain open because our unexplored universe is expanding in space and time. My hope and belief is that there will never come a time when we shall say, ‘We are done’.”

At the symposium on Physiology and Pathophysiology 2023, we shall undoubtedly hear about many exciting new and advancing frontiers in our subject. Physiologists are beginning to address very ambitious goals such as understanding the basic physiology of learning and language as well as attempting to make real progress on the pathophysiology of cardio-vascular diseases, pancreatic cancer, and dementia, to name just a few examples. Given that many of the techniques used in apparently different branches of physiology are remarkably similar, physiologists have much in common and therefore have a lot to learn from each other. We are undoubtedly better at analysis than synthesis, so one of the aims of the conference is to focus on putting things together rather than pulling them apart. Communication is key, and both direct talks and publications play important roles. We therefore plan to publish the papers relating to the invited presentations in *Function*, and we shall compile them into a virtual issue. I am confident that it will not only be an impressive showcase for contemporary physiology but also point to many issues that still need to be addressed. Physiology is alive and advancing!
